# Linking energy availability, movement and sociality in a wild primate (*Papio ursinus*)

**DOI:** 10.1098/rstb.2022.0466

**Published:** 2024-10-28

**Authors:** Ines Fürtbauer, Chloe Shergold, Charlotte Christensen, Anna M. Bracken, Michael Heistermann, Marina Papadopoulou, M. Justin O'Riain, Andrew J. King

**Affiliations:** ^1^ Biosciences, Faculty of Science and Engineering, Swansea University, Swansea SA2 8PP, UK; ^2^ Department of Evolutionary Biology and Environmental Science, University of Zurich, Zurich 8057, Switzerland; ^3^ School of Biodiversity, One Health and Veterinary Medicine, University of Glasgow, Glasgow G12 8QQ, UK; ^4^ Endocrinology Laboratory, German Primate Center, Göttingen 37077, Germany; ^5^ Institute for Communities and Wildlife in Africa, Biological Sciences Department, University of Cape Town, Cape Town, Rondebosch 7701, South Africa

**Keywords:** social ageing, energy, movement, thyroid hormones, grooming, baboons

## Abstract

Proximate mechanisms of ‘social ageing’, i.e. shifts in social activity and narrowing of social networks, are understudied. It is proposed that energetic deficiencies (which are often seen in older individuals) may restrict movement and, in turn, sociality, but empirical tests of these intermediary mechanisms are lacking. Here, we study wild chacma baboons (*Papio ursinus*), combining measures of faecal triiodothyronine (fT3), a non-invasive proxy for energy availability, high-resolution GPS data (movement and social proximity) and accelerometry (social grooming durations). Higher (individual mean-centred) fT3 was associated with increased residency time (i.e. remaining in the same area longer), which, in turn, was positively related to social opportunities (i.e. close physical proximity). Individuals with more frequent social opportunities received more grooming, whereas for grooming given, fT3 moderated this effect, suggesting an energetic cost of giving grooming. While our results support the spirit of the energetic deficiencies hypothesis, the directionality of the relationship between energy availability and movement is unexpected and suggests that lower-energy individuals may use strategies to reduce the costs of intermittent locomotion. Thus, future work should consider whether age-related declines in sociality may be a by-product of a strategy to conserve energy.

This article is part of the discussion meeting issue ‘Understanding age and society using natural populations’.

## 1. Introduction

For group-living species, social connections represent a means to cope with socio-environmental challenges [1–3]. While all individuals in a group can benefit from advantages (e.g. social information; [4,5]), how well an individual is socially integrated into their group can vary, altering the costs and benefits of sociality [6–10] and impacting individuals’ fitness [11–13]. Only recently, however, research into sociality and its adaptive consequences has acknowledged that social connections are not stable across the lifespan and likely change in their form and function as individuals transition across life-history stages [14,15]. This phenomenon—referred to as social ageing—predicts age-related changes in social behaviour and is crucial to exploring evolutionary perspectives of ageing [16].

Understanding the process of ageing is critical given that humans are living into old age at an unprecedented rate [17,18]. ‘Successful’ ageing is not solely reliant on physical health but is inclusive of psychological and social wellbeing [19–21]. Characteristically, old age in people is accompanied by a shrinking social network, an overall decrease in social behaviour and a higher degree of social selectivity that sees old-aged individuals focusing on few significant relations and emotionally meaningful interactions [22–25]. However, old-aged people also have more social experience and are more skilled at solving conflict than younger adults [26], leading to a complex picture of social ageing where variation along these facets may shape an individual’s ageing experience and to this extent, how successful it is [19].

Social ageing is not unique to humans, and non-human animals—in particular, non-human primates—offer a valuable comparative insight into social ageing in the absence of cultural conventions and an awareness of a limited lifespan as seen in humans (‘socio-emotional selectivity theory’; [27,28]). Non-human primates are usually highly gregarious and have an extended period of old age [29–31], and functionally important social behaviours can be quantified through social grooming [32–34]. Similar to humans, age-related changes in social behaviour in non-human primates include a reduction in time or frequency of social grooming (*Macaca sylvanus* [35,36]; *Macaca fuscata* [37]; *Macaca mulatta* [38]; *Presbytis comata* [39]; *Sapajus apella* [40]), a reduction in close spatial associations (*M. sylvanus* [35,36]; *Macaca thibetana* [41]), an increase in avoidance behaviour (*Macaca arctoides* & *M. fuscata* [42]), sex-dependent changes in receiving aggression (*M. thibetana* [41]) and changes to social networks (*M. sylvanus* [43]; *M. mulatta* [44,45]; *Pan troglodytes* [46]) and social partner choice (*M. mulatta* [38]).

The decline in physiological function that occurs with ageing decreases survival probability and reproduction prospects in wild animals [47–51]. Decreased body mass, reduced energetic intake and decreased resting metabolic rate result in a decline in energy availability, and thus in energy expenditure and activity levels [52–55]. It is a reasonable assumption then that old-aged monkeys that are less active spend more time resting and performing fewer energetically demanding behaviours [35,39,56] because of energetic deficiencies [52,56,57]. Importantly, these individuals are less integrated into their social group, i.e. have fewer grooming partners and spend more time in social isolation [35,36,39,42]. Energetic deficiencies have therefore been proposed as one major driver of social ageing [14,15,36,58]. In particular, ‘energetic deficiencies are expected to restrict an individual’s movement and therefore their ability to socially engage or the likelihood of others coming into social contact, leading to reductions in the quality and quantity of social relationships’ [15, p. 6]. However, monitoring energetic condition and quantifying movement and sociality in wild animals pose major methodological challenges and formal tests of the ‘energetic deficiencies hypothesis’ (sensu [15]) are therefore lacking.

Here, we study wild chacma baboons (*Papio ursinus*) and examine the links between energy availability, movement and sociality, which are proposed as intermediary mechanisms underpinning the 'energetic deficiencies hypothesis' [15]. We overcome the limitations of previous research by combining high-resolution bio-logging data (GPS, accelerometry) to quantify broad- and fine-scale movement, physical proximity and individual sociality (grooming given and received; [34]) and non-invasive monitoring of faecal triiodothyronine (fT3) concentrations as a proxy for energy availability [59,60]. Thyroid hormones, triiodothyronine (T3) and its prohormone thyroxine (T4), regulate energy metabolism and can be used as markers of energy availability [59,61,62]. Moreover, T3—the biologically active hormone—significantly declines with age in humans and non-human mammals (reviewed by Behringer *et al*. [62]). In line with the ‘energetic deficiencies hypothesis’ (see above), we test the following linked mechanisms: (i) energy availability affects movement; (ii) movement affects social opportunities; and (iii) social opportunities affect grooming interactions. Our study focuses on spatial movement (and the associated likelihood of coming into social contact); however, because energy availability may also restrict individuals’ ability to socially engage (see Siracusa *et al*. [15]), we also test for a more direct effect of energy availability on (giving) grooming.

## 2. Methods

### (a) Study site and animals

The study was conducted on a troop of fully habituated wild chacma baboons in Da Gama Park, Western Cape, South Africa (−34.15562° N, 18.39858° E). The troop consisted of approximately 50 individuals, including *n* = 21 adults. Most adult baboons (*n* = 16) were fitted with tracking collars (for full details see Christensen *et al.* and Bracken *et al*. [34,63]). Collars were built at Swansea University and included a GPS and tri-axial accelerometer and were approved by Swansea University’s Ethics Committee (IP-1315-5) and local authorities (Cape Nature, permit number: CN44-59-6527; SANparks, permit number: CRC/2018-2019/008-2018/V1). Dominance rank was determined previously, based on the outcome of directly observed dyadic agonistic interactions and using the I&SI method and R packages ‘aniDom’ [64] and ‘compete’ [65]. Ranks were standardized between 0 (lowest) and 1 (highest) [63,66].

### (b) Faecal sample collection, extraction and hormone analysis

Faecal samples were collected from all adult, individually identifiable baboons (*n* = 19 females and *n* = 2 males) between June and November 2018 (for details see Christensen [67]). Samples were collected opportunistically during all-day follows and kept on cool blocks in a cool bag before being frozen at −20°C at the research house until further processing. Samples were freeze-dried at the University of Cape Town, and dried samples were shipped to Swansea University (CITES export permit no: 208683; APHA import authorization no: ITIMP18.1181) for faecal hormone analysis. Hormone extraction procedures are described in detail by Fürtbauer *et al*. [66].

Faecal samples were previously analysed for progestogen metabolites to assess ovarian activity and female reproductive state (for details see Fürtbauer e*t al*. [66]). Here, the same samples (*n* = 641) were analysed for fT3 as a proxy for energy availability [59], using a commercial ELISA kit for total T3 (IBL RE55251). The assay has been used successfully in several species to quantify faecal and urinary T3 (e.g. [60,68,69]). 300 µl of faecal extract were evaporated at 38°C under a stream of nitrogen and reconstituted with 250 µl assay buffer (Standard A; IBL RE55256). Reconstituted sample extracts were then assayed according to the manufacturer’s instructions. Inter-assay coefficients of variation calculated from replicate determinations of low- and high-value quality controls included on each assay plate (*n* = 16) were 9.1% (low) and 6.0% (high). The sensitivity of the assay was <0.1 ng ml^−1^. fT3 concentrations are expressed as ng g^−1^ faecal dry weight.

To validate our proxy of energy availability and to demonstrate that fT3 levels are biologically meaningful in our study context, i.e. that older individuals have lower energy availability indicated by lower fT3, we tested for a correlation between estimated age and average fT3 levels in our sample. Veterinary tooth wear examination during collar fitting suggested that study animals’ ages ranged from approximately 4 to 18 years. Owing to the absence of exact ages, the authors leading fieldwork (C.C. and A.M.B.) ranked all adult individuals from youngest to oldest based on physical features (i.e. scars, elongated/discoloured nipples/breast, ‘looser’ appearance of skin) during five months of direct observation of the troop. The two independent age rankings were significantly positively correlated (Spearman’s *ρ* = 0.74, *p* < 0.001, *n* = 21). Standardized mean age ranks (between 0 and 1, with 0 being the youngest and 1 the oldest individual; function *range01* in the R package 'funModeling'; [70]) and mean fT3 concentrations were significantly negatively correlated (Spearman’s *ρ* = −0.53, *p* < 0.014, *n* = 21), confirming that older individuals had lower average levels of fT3, as shown across a range of species (reviewed by Behringer *et al*. [62]). Note that we do not test for a direct relationship between age and sociality because our study concerns proposed intermediary mechanisms, i.e. that energy availability affects movement and movement affects sociality (see above).

### (c) Movement metrics

To test the effect of energy availability on movement, we used high-resolution GPS data available for *n* = 11 females and *n* = 2 males [63,71] to provide four movement metrics of different spatial and temporal scales for each baboon, for each day: (i) total daily travel distance, which is a simple but revealing measure of an animal’s broad-scale movement (e.g. [72,73]), and median daily (ii) step length, (iii) sinuosity and (iv) residence time. (ii–iv) are fine-scale metrics commonly used to describe inter-individual differences in movement (e.g. [74]) and they provide information on how quickly individuals are moving (step length; the distance between time steps), the directness of their trajectory (sinuosity; calculated as a function of the mean cosine of turning angles) and how long they spend in one area (residence time; time spent inside the radius of its mean step length centred on its GPS position without leaving the radius for more than a specified cut-off time). We calculated residence time for trajectories with a minimum of five consecutive GPS fixes (i.e. a ‘path-segment’ of >5 min, while setting the cut-off time at 5 min; [75]). Because our study subjects spend a small proportion of each day in urban space [71] and their movement is very different in this environment [75], we used these metrics (ii–iv) calculated for natural spaces only. For a full description of (ii–iv), see Bracken *et al*. [75]. Because energy availability is expected to restrict movement, we expected positive correlations between fT3 levels and movement metrics (i–iii) and a negative correlation between fT3 and (iv).

### (d) Social opportunities and grooming

The likelihood of coming into social contact with others is suggested to limit the sociality of old-aged individuals [15]. We therefore conceptualized a ‘social opportunity event’ where an individual’s spatial position would have allowed for social interactions to occur, i.e. close physical proximity to another adult (and collared) individual, defined as ≤2 m apart. To calculate social opportunity events, we extracted nearest neighbour information from GPS data using the R package ‘SwaRm’ [76] and identified all times when an individual had a nearest neighbour under or equal to a threshold of two meters. If a secondary event occurred within 2 s of the first—between the same two individuals—it was counted as a single opportunity event rather than a separate event. The frequencies of social opportunity events per individual and day were used in subsequent analyses. Given that social opportunity events were dependent on GPS data, i.e. the number of active collars, we used a subset of data where at least 10 collars were active since this is where we have found social network metrics to be robust [71], and we additionally control for the number of active collars in our models (see below).

Social grooming was previously identified using machine learning (random forest models) trained on tri-axial acceleration data from collared individuals (for full description see Christensen *et al*. [34]). Receiving and giving grooming were identified with high precision (91 and 81%) and recall (79 and 87%). Here, we use the total duration of giving and receiving grooming (minutes) between sunrise and sunset to avoid potential misclassification and overestimation of receiving grooming during the night [34].

### (e) Statistical analysis

All analyses were conducted in R v. 4.2.3 [77] and RStudio ([78]; v.2023.03.1). To test intermediary mechanisms underlying the ‘energetic deficiencies hypothesis’ (*sensu* [15]), we ran linear mixed models (LMMs) using the R package 'lmerTest' [79]. The significance level was set to *p* < 0.05. Where appropriate, response and predictor variables were transformed to achieve normality (see below and electronic supplementary material for details). All continuous predictor variables were *z*-transformed [80]. Model assumptions were checked using the package 'performance' [81]. To test for collinearity, variance inflation factors (VIFs) were examined using the package 'car' [82]. The maximum VIF across all models was 1.3, suggesting no collinearity issues [83]. The significance of the full models as compared to the null models (only including random effects and control variables) was established using likelihood ratio tests (R function *anova*).

#### (i) Energy availability and movement

To test whether energy availability affects movement, we ran four LMMs (LMM1–LMM4; see electronic supplementary material for LMMs 1–3 and table 1 for LMM4) with each of the four movement metrics (daily total travel distance, median daily step length, sinuosity, and residence time) included as response variables (step length, sinuosity and residence time were log-transformed). To test whether changes in energy availability drive changes in movement within individuals on a day-to-day basis (within-individual effect), while also considering broader patterns across individuals (between-individual effect), we split our predictor (fT3) into two variables, representing a different source of variance, i.e. each individual’s average fT3 (mean fT3) and the deviation of each measurement from the individual’s mean (mean-centred fT3), and included both of these as continuous fixed effects. If more than one fT3 measure was available per individual/day, mean daily fT3 measures were used. To account for the time lag for hormone excretion into faeces, we imposed a 48 h time lag on the fT3 data (e.g. [84]). To account for the potential effects of reproductive state on movement [85], reproductive state (i.e. acyclic, cyclic, pregnant, lactating and male) was included as a categorical fixed effect. Baboon ID and date were included as random effects to account for individual and daily variation. Random slopes were fitted for mean-centred fT3 within Baboon ID. Data were available for *n* = 13 individuals (LMM1: *n* = 163 observations; LMMs 2–4: *n* = 175 observations).

**Table 1.** Summary of LMMs. All models included Baboon ID and date as random effects (for details on random slopes, see §2*e*). Significant effects are highlighted in bold. For full model outputs and results of LMMs 1–3, see electronic supplementary material. *N* = number of individuals; *n* = number of observations. See §2*e* for more details.

mechanism	model	response	*N*	est ± SE	*t*-value	*p-* value	*N*	*n*
energy availability affects movement	LMM4	residence time (minutes)	fT3 (mean)	0.00 ± 0.04	0.11	0.909	13	175
			fT3 (mean-centred)	0.12 ± 0.04	3.04	**0.003**		
			reproductive state^a^			0.067		
movement affects social opportunities	LMM5	social opportunity (daily frequency)	residence time	1.01 ± 0.24	4.21	**<0.001**	11	367
			daylength	−0.26 ± 0.28	−0.91	0.362		
			reproductive state^a^			**0.024**		
			dominance rank	1.50 ± 0.26	5.7	**<0.001**		
			number of active collars^b^	0.50 ± 0.30	1.67	0.096		
social opportunities affect grooming	LMM6	receiving grooming (minutes)	social opportunity	0.74 ± 0.16	4.57	**<0.001**	8	301
			daylength	0.21 ± 0.16	1.35	0.177		
			reproductive state^a^			0.087		
			dominance rank	−0.80 ± 0.52	−1.53	0.127		
	LMM7	giving grooming (minutes)	social opportunity × fT3	0.56 ± 0.17	3.30	**0.001**	8	103
			daylength	0.37 ± 0.22	1.67	0.098		
			reproductive state^a^			0.589		
			dominance rank	0.82 ± 0.67	1.22	0.227		

^a^ Overall effect of reproductive state

^b^ Days where 10 or more collars were active are included in this analysis

#### (ii) Movement and social opportunities

Based on the results of LMM1–LMM4, residence time, which was significantly predicted by energy availability (i.e. mean-centred fT3), was carried forward to test whether residence time (log- and *z*-transformed) affects (square-root transformed) daily social opportunity frequencies (LMM5; table 1). Because baboon social interactions and structure are predominantly female-based, we only include females in this (and subsequent) analysis. Since social interactions can change across female reproductive state [86,87], reproductive state was included as a categorical fixed effect. Because individuals are expected to have more frequent social opportunities on longer days, we included daylength (hours) as a continuous fixed effect. Because higher-ranked individuals are more central in association networks [63], we also included dominance rank as a fixed effect. We also added the number of active collars (individuals in the sample at a given time) as a control variable. Baboon ID and date were included as random effects, and random slopes were fitted for residence time within Baboon ID. Data were available for *n* = 11 females (*n* = 367 observations).

#### (iii) Social opportunities and grooming interactions

To test whether social opportunities affect social interactions (i.e. social grooming), we tested whether daily social opportunity event frequency (*z*-transformed) predicted either grooming received (square-root transformed; LMM6; table 1) or grooming given (square-root transformed; LMM7; table 1). Giving and receiving grooming were considered separately because (i) social ageing effects are not equal across grooming given and received (e.g. [35,43]) and (ii) giving grooming is an active and therefore likely more energetically demanding behaviour, while being groomed is more passive. We examined the impact of energy availability (fT3) on giving grooming by including an interaction term between fT3 and the frequency of social opportunities (LMM7). Reproductive state, dominance rank and daylength were included as fixed effects, and Baboon ID and date were included as random effects. Random slopes were fitted for social opportunities within Baboon ID. Matched GPS and grooming data were available for *n* = 8 females (LMM6: *n* = 301 observations; LMM7: *n* = 103 observations).

## 3. Results

**Figure 1.**
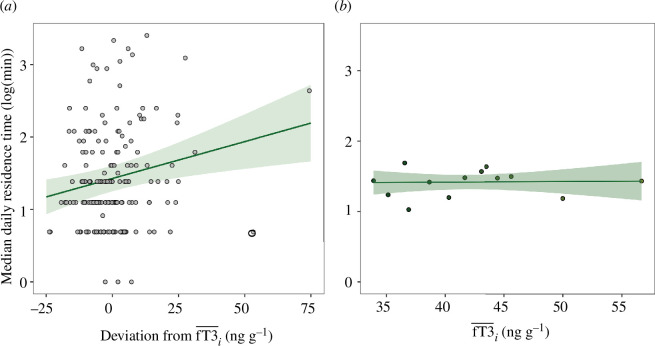
(*a*) Within- and (*b*) between-individual effect of energy availability (fT3) on median daily residence time. The shaded green areas indicate 95% confidence intervals around the predicted values (solid green lines). Note that mean and individual mean-centred fT3 were *z*-transformed for statistical analysis.

**Figure 2.**
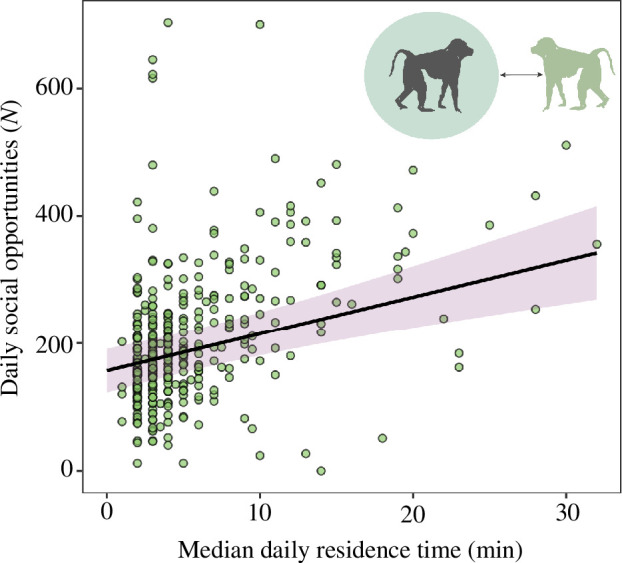
Effect of median daily residence time (i.e. the time an individual spends inside the radius of its mean step length centred on its GPS position) on daily social opportunities (i.e. the frequency of coming within 2 m proximity of others). The shaded pink area indicates 95% confidence intervals around the predicted values (solid black line). Note that daily social opportunities were square-root transformed, and residence time was log- and *z*-transformed for statistical analysis.

**Figure 3.**
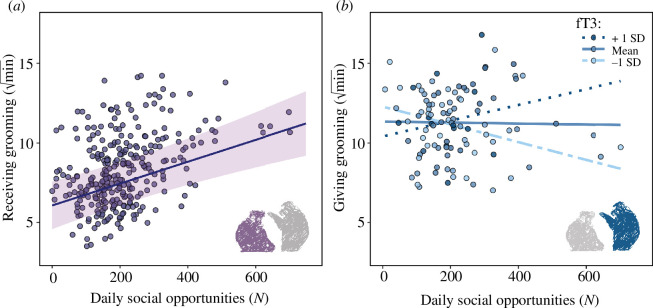
Effect of daily social opportunities on accelerometer-derived total (*a*) receiving and (*b*) giving grooming between sunrise and sunset. For giving grooming (*b*), an interaction was fitted between fT3 and social opportunities. Note that daily social opportunities and fT3 were *z*-transformed for statistical analysis.

Full versus null model comparisons for LMMs 1–3 (testing for effects of fT3 on total distance travelled, step length and sinuosity) were non-significant (smallest *p* = 0.309; for model outputs, see electronic supplementary material). The model testing for an effect of fT3 on residence time was marginally significantly different from the null model (LMM4: *df* = 4, *χ*² = 8.90, *p* = 0.06). Individual mean-centred fT3 was significantly positively associated with residence time (LMM4: estimate ± SE = 0.12 ± 0.04, *t* = 3.04, *p* = 0.003; table 1 and figure 1*a*), indicating that individuals remain in the same area for longer when their energy availability is higher. No between-individual effect of fT3 on residence time was found (LMM4: estimate ± SE = 0.00 ± 0.04, *t* = 0.11, *p* = 0.909; table 1 and figure 1*b*). The model testing for the effect of residence time on social opportunities was significantly different from the null model (LMM5: *df* = 3, *χ*² = 30.86, *p* < 0.001). Residence time was significantly positively associated with daily social opportunity event frequency (LMM5: estimate ± SE = 1.01 ± 0.24, *t* = 4.21, *p* < 0.001; table 1 and figure 2), indicating that remaining in the same area for longer increases the likelihood of coming into social contact with others. Higher-ranking individuals had more social opportunities (LMM5: estimate ± SE = 1.50 ± 0.26, *t* = 5.70, *p* < 0.001; table 1). The models testing for an effect of social opportunities on grooming were significantly different from the null models (LMM6: *df* = 3, *χ*² = 49.72, *p* < 0.001; LMM7: *df* = 3, *χ*² = 16.63, *p* < 0.001). Daily social opportunity frequency was significantly positively associated with time spent receiving grooming (LMM6: estimate ± SE = 0.74 ± 0.16, *t* = 4.57, *p* < 0.001; table 1 and figure 3*a*). Time spent giving grooming was predicted by a significant interaction between fT3 and daily social opportunity frequency (LMM7: estimate ± SE = 0.56 ± 0.17, *t* = 3.30, *p* = 0.001; table 1 and figure 3*b*), indicating that social opportunities are associated with more grooming given when energy availability is higher (for full model outputs, see electronic supplementary material).

## 4. Discussion

Shifts in social activity and interactions are often seen in non-human primates as individuals age [14–16,35–37,39–43]. However, proximate mechanisms of social ageing remain understudied [14,15]. Energetic deficiencies in older age have been proposed as one major mechanism that restricts movement and, in turn, social opportunities and interactions [15]. Systematic investigations of these intermediary mechanisms are lacking owing to methodological constraints. Here, we monitored fT3, a non-invasive proxy for energy availability [59,61,62], for individuals for which we have high-resolution data detailing their spatial movement and social interactions (from GPS and accelerometers), allowing us to test whether (i) energy availability affects movement, (ii) movement affects social opportunities, and (iii) social opportunities affect grooming interactions. Below we discuss the results of these tests and outline broader implications/considerations for our understanding of (energetic) drivers of social ageing in (wild) non-human primates.

### (a) Linking energy availability and movement

Energetic deficiencies in older age are predicted to restrict movement, and older monkeys have been found to rest more frequently [42] and spend less time engaging in more energy-demanding behaviours like running, jumping or climbing [35,36] than younger individuals. Here, we found that energy availability (fT3) did not significantly predict how far (total daily distance), how fast (step length) or how tortuously (sinuosity) baboons moved (see electronic supplementary material). Perhaps this is unsurprising given that chacma baboons show high synchrony in activity and they travel collectively [63,86]. Indeed, group-living animals make locomotor compromises so that they can travel together, reducing variance in travel via conformity effects [88,89], which results in similar travel trajectories and distances travelled over a day [73,90]. The fact that we see the lowest coefficients of variation for distance travelled and sinuosity in our data supports this interpretation (see electronic supplementary material). Highly cohesive groups like baboons therefore contrast with species that have more fluid social systems, where age-related changes in spatial behaviours are linked to changes in observed social connectedness [58].

Although we do not find evidence for restricted spatial movement owing to energetic deficiencies, we show that higher energy availability (individual mean-centred fT3) was associated with longer residence times (figure 1*a*). At first glance, this finding speaks against the idea that having less energy ‘restricts’ movement, not least because the phrasing ‘residence time’ suggests individuals are stationary, but this need not be the case. Higher residence times can also result from individuals moving slowly and/or tortuously [91,92], since both would result in small displacements. This is what we see in our dataset, where residence time is positively correlated with sinuosity and negatively with step length (see electronic supplementary material LMMS1 and electronic supplementary material, figure S2). Importantly, a more tortuous path indicates that individuals are turning more, which is energetically more costly [93], and when moving at low speed—and close to 0—(which results in longer residence times; see electronic supplementary material, figure S2B), fixed energetic costs are greater than the costs incurred when moving at slightly higher speeds [93]. Longer residence times are also likely to be associated with more frequent acceleration and deceleration, i.e. more intermittent movement, which also incur expenditure costs [94]. Individuals with lower energy availability may therefore reduce these energetic costs by decreasing their residence times and moving more continuously. Thus, age-related declines in sociality seen in other research could be by-products of an active strategy to conserve energy rather than, or in addition to, a passive consequence of restricted movement. One would expect to see such a strategy in systems like baboons where individuals stay as a cohesive group and can travel over long distances each day [72].

It is also possible that individuals with higher energy availability have longer residence times because residence times are related to some aspect of their foraging ecology. In general, variation in residence times can be interpreted in two ways, depending on the spatial heterogeneity of resource availability [95]. When resources are distributed unevenly, longer residence times could indicate higher resource availability. While fT3 is positively associated with food availability [96] and food intake [97], feeding time in Barbary macaques (*M. sylvanus*)—which is inversely correlated with food availability—is negatively correlated with fT3 [60]. In our study, variation in residence times is unlikely to be associated with foraging since the dominant natural vegetation (and forage) type is ‘fynbos’ [98], which is relatively evenly distributed across the troop’s home range [63] and of low quality [99], and we exclude the short periods of time when baboons are in urban space, where resource availability is heterogeneous [100]. In environments with more even resource distribution—like our study—variation in residence times is often explained by differences in how individuals explore their environment, with more exploration of the local environment resulting in higher residence times [95].

### (b) Linking movement and sociality

We further tested whether movement affects social opportunities, i.e. the likelihood of coming into close contact with others [15]. We show that longer residence times (which are associated with higher energy availability; see above) predicted more frequent social opportunities (figure 2). This means that baboons that remain in an area for longer (where they may be stationary, slow-moving or turning lots within a small area; see above) have more frequent social opportunities (i.e. close physical proximity to others). Those individuals with more social opportunities received more grooming, as indicated by accelerometer-derived total durations of grooming [34] (figure 3*a*). We also found that the effect of physical proximity on giving grooming was moderated by fT3—in other words, social opportunities were positively associated with giving grooming only when energy availability (fT3) was high (figure 3*b*). This suggests that there is an energetic cost associated with giving grooming (see also Christensen *et al*. [101]) and could possibly explain why social ageing effects seen in other work appear to be more pronounced for giving grooming compared with receiving grooming [43,102].

The links between energy-dependent movement and sociality that we find here highlight a need to further investigate how changes in spatial behaviours are linked to changes in observed social behaviours (e.g. [58]). Our investigation is only possible because of the high-resolution movement and behavioural data that the tracking collars provide. Indeed, we estimate social opportunities and the total duration of giving and receiving grooming between sunrise and sunset for all collared individuals. Such data are impossible to collect using direct observations. So, while we do have a small error in our calculation of the position of individuals [71] and classification of behaviour [34] and we do not have data for all adult individuals [63], our dataset and analyses nevertheless represent a step-change in our ability to test and extend current theory. For example, given that residence time predicts social opportunities (see above), we would also expect individuals with shorter residence times to encounter fewer individuals, leading to interactions with fewer partners. It is therefore possible that lower-energy individuals may also be constrained in who they interact with (‘constrained sociality’) rather than selectively interacting with fewer/certain individuals (‘selective sociality’; e.g. [35,38,43]). Future modelling of movement dynamics will allow us to make more precise predictions about the links between movement, social opportunities and both the quality and quantity of social relationships for individuals who live in social systems that vary in their degree of coordinated movement and synchrony in activity [103]. For example, if grooming networks are influenced by social opportunity, which is lower when adopting a presumably more energy-saving movement strategy, then lower-energy individuals are predicted to have fewer grooming partners, reduced grooming reciprocity and to occupy peripheral positions within grooming networks.

### (c) Energetic drivers of social ageing in (wild) non-human primates

Rooted in evidence of age-related decreases in physical activity across humans and non-human animals (for reviews, see Ingram [56] and Manini [57]), energetic deficiencies—and associated changes in movement—have been proposed as a driver of social ageing [15]. In this study, we have combined a non-invasive proxy of energy availability (fT3) and high-resolution movement data (accelerometry, GPS) to present the first systematic test of the intermediary mechanisms that underpin the 'energetic deficiencies hypothesis' for social ageing [15]. On days with higher energy availability, individuals showed increased residency time (i.e. remaining in the same location longer either owing to moving more slowly or in a more tortuous way), which, in turn, was positively related to social interactions. Therefore, while our results support the spirit of the 'energetic deficiencies hypothesis'—i.e. social senescence could emerge from changes in movement related to energetic conditions [15]—we do not find that having less energy ‘restricts’ spatial movement.

The absence of a between-individual effect of energy availability on movement in our dataset (figure 1*b* and electronic supplementary material, figure S1) does not rule out the possibility of a population-level effect of energy on movement/sociality across ages. First, female reproductive state—which affects T3 and the relationship between activity and T3 (e.g. [97])—may be a confounding effect. Second, we analysed relatively few individuals (*n* = 13), and third, because estimated ages in our study ranged from approximately 4 to 18 years (based on veterinary tooth wear examination), it is unlikely we have sampled ‘very old’ individuals and therefore may have too narrow a range of fT3 to detect a between-individual effect. Indeed, most of the evidence for age-related declines in locomotion and/or sociality in cercopithecines comes from studies of captive or provisioned free-ranging populations with no natural predators (e.g. [35,36,38,43,45]). Food provisioning and the absence of predators likely increase life expectancy, and thus the proportion of (very) old individuals in such groups (age of up to 30 years [35,36,38,43]; but note that this maximum age has also been reported for wild macaques [102]). This makes such systems an excellent analogue model for human social ageing work. Indeed, agent-based models predict that age distribution affects changes in social network structure [45]. Specifically, for populations with a smaller range of age distributions, changes in networks may not be detectable [45]. So while the intermediary mechanisms (links between energy, movement and sociality) proposed by the 'energetic deficiencies hypothesis' are expected to be a general phenomenon (and therefore apply across ages), we may not have ‘old enough’ individuals to see variation in energetic deficiencies that translate into substantial changes (i.e. restrictions) in movement. For example, studies of Barbary macaques (*M. sylvanus*) show prominent age effects on movement for the very oldest individuals, but more variation for individuals in the rest of the age range [36,43]. Furthermore, because wild primates travel over long distances as cohesive and coordinated groups (compared to captive or provisioned groups that typically have restricted space), it may be impossible for older individuals to move less—they simply would not keep up (see electronic supplementary material for coefficients of variation in movement metrics).

Overall, our study has revealed a more subtle energy–movement–sociality mechanism, whereby lower-energy individuals move differently and presumably more energy-efficiently (see above), affecting social opportunities and grooming. Our findings therefore highlight further potential ways in which age-dependent declines in energy availability may affect movement and, in turn, sociality. We suggest that age-related changes in sociality may be a by-product of a strategy to conserve energy, which can be tested in future work and other contexts and systems.

## Data Availability

The data are available online at [104].
